# Can Chinese herbal medicine offer feasible solutions for newly diagnosed esophageal cancer patients with malnutrition? a multi-institutional real-world study

**DOI:** 10.3389/fphar.2024.1364318

**Published:** 2024-05-24

**Authors:** Yi-Chin Lu, Liang-Wei Tseng, Chiao-En Wu, Ching-Wei Yang, Tsung-Hsien Yang, Hsing-Yu Chen

**Affiliations:** ^1^ Division of Chinese Internal Medicine, Center for Traditional Chinese Medicine, Chang Gung Memorial Hospital, Taoyuan, Taiwan; ^2^ Division of Chinese Acupuncture and Traumatology, Center of Traditional Chinese Medicine, Chang Gung Memorial Hospital, Taoyuan, Taiwan; ^3^ Division of Haematology-Oncology, Department of Internal Medicine, Chang Gung Memorial Hospital at Linkou, Chang Gung University College of Medicine, Taoyuan, Taiwan; ^4^ School of Traditional Chinese Medicine, College of Medicine, Chang Gung University, Taoyuan, Taiwan; ^5^ Graduate Institute of Clinical Medical Sciences, College of Medicine, Chang Gung University, Taoyuan, Taiwan

**Keywords:** Chinese herbal medicine, Chinese herbal medicine network, pharmacology network, esophageal cancer with malnutrition, survival analysis

## Abstract

**Background:**

Esophageal cancer (EC) is a major cause of cancer-related mortality in Taiwan and globally. Patients with EC are highly prone to malnutrition, which adversely affects their prognosis. While Chinese herbal medicine (CHM) is commonly used alongside conventional anti-cancer treatments, its long-term impact on EC patients with malnutrition remains unclear.

**Methods:**

This study utilized a multi-center cohort from the Chang Gung Research Database, focusing on the long-term outcomes of CHM in EC patients with malnutrition between 1 January 2001, and 31 December 2018. Patients were monitored for up to 5 years or until death. Overall survival (OS) rates were calculated using the Kaplan-Meier method. Overlap weighting and landmark analysis were employed to address confounding and immortal time biases. Additionally, the study analyzed prescription data using a CHM network to identify key CHMs for EC with malnutrition, and potential molecular pathways were investigated using the Reactome database.

**Results:**

EC patients with malnutrition who used CHM had a higher 5-year OS compared with nonusers (22.5% vs. 9% without overlap weighting; 24.3% vs. 13.3% with overlap weighting; log-rank test: *p* = 0.006 and 0.016, respectively). The median OS of CHM users was significantly longer than that of nonusers (19.8 vs. 12.9 months, respectively). Hazard ratio (HR) analysis showed a 31% reduction in all-cause mortality risk for CHM users compared with nonusers (HR: 0.69, 95% confidence interval: 0.50–0.94, *p* = 0.019). We also examined 665 prescriptions involving 306 CHM, with *Hedyotis diffusa* Willd. exhibiting the highest frequency of use. A CHM network was created to determine the primary CHMs and their combinations. The identified CHMs were associated with the regulation of immune and metabolic pathways, particularly in areas related to immune modulation, anti-cancer cachexia, promotion of digestion, and anti-tumor activity.

**Conclusion:**

The results of this study suggest a correlation between CHM use and improved clinical outcomes in EC patients with malnutrition. The analysis identified core CHMs and combinations of formulations that play a crucial role in immunomodulation and metabolic regulation. These findings lay the groundwork for more extensive research on the use of CHM for the management of malnutrition in patients with EC.

## 1 Introduction

Esophageal cancer (EC) remains among the top 10 leading causes of cancer-related mortality (Abnet et al., 2018). Globally, it is estimated that 45,000 cases of EC occurred in 2012, with incidence rates of 5.9 per 100,000 (Arnold et al., 2015). The treatment for EC has evolved from traditional approaches such as surgery and chemotherapy to now include targeted therapies and immunotherapies, improving patient outcomes and laying the foundation for precision medicine (Yang et al., 2020). Despite a recent decline in overall mortality rates, EC continues to demonstrate a disconcertingly low survival rate, estimated at 15%–25% (Domper Arnal et al., 2015; Cheng et al., 2018). Malnutrition is a critical factor and significantly influences the prognosis of patients with cancer (Bossi et al., 2021). Patients with EC are particularly susceptible to malnutrition, with prevalence rates ranging 29.7%–88% (Chen et al., 2018; Cao et al., 2021). Previous research has established a link between the nutritional status of patients with EC and their prognostic outcomes, especially for those in the terminal stages (Li et al., 2020; Okadome et al., 2020; Qiu et al., 2020; Jiang et al., 2021). The nutritional status profoundly affects postoperative outcomes and complications (Takeuchi et al., 2014; Yoshida et al., 2016; Horinouchi et al., 2022). Prior investigations have also delved into the implications of nutritional interventions in EC management (Chen et al., 2018; Qiu et al., 2020).

Considering the heightened risk of malnutrition in EC, assessment of the nutritional status of such patients is essential. Commonly employed markers for this evaluation include serum albumin, total lymphocyte count (TLC), and the Prognostic Nutritional Index (PNI) (Okadome et al., 2020). The PNI is derived from albumin levels and TLC. It offers a convenient method for risk stratification, identifying patients with moderate to severe malnutrition who are more susceptible to complications, affirming its role as an important prognostic biomarker (Okadome et al., 2020). A research indicated that PNI has a moderate predictive capacity for overall survival (OS), disease-free survival, and cancer-specific survival in patients with EC (Jiang et al., 2021).

Chinese herbal medicine (CHM) has been widely used as an adjunctive therapy among patients with diverse types of cancer. A well-established effect of CHM is its potential to mitigate gastrointestinal side effects and augment nutritional intake (Qi et al., 2015; Zhang et al., 2021; Wu et al., 2022). Research conducted thus far has primarily been focused on particular stages of EC. A retrospective clinical study has indicated that administration of a specified CHM formula concomitant with adjuvant chemoradiotherapy could enhance progression-free survival and OS in patients with stage II and III EC (Chang et al., 2017). Another study, which combined CHM with chemoradiotherapy for patients with advanced-stage EC, found a diminished frequency and severity of radiation-induced lung injury, better clinical outcomes, and an improved quality of life (Cui et al., 2016). Post esophagectomy, the integration of CHM has been correlated with improved 3-year OS rates, superior quality of life, and enhanced immune responses (Lu et al., 2006). Our prior research revealed that the administration of CHM appears both safe and advantageous for patients with stage IV EC, demonstrating increased 5-year OS rates (Chen et al., 2022b). Nonetheless, the effects of CHM on the OS metrics across all EC patient groups, particularly the malnourished subset, are yet to be fully investigated. While earlier studies postulated a possible role for CHM in hastening gastrointestinal recovery post esophagectomy, its influence on survival metrics remains unresolved (Hu et al., 2011).

This study aims to evaluate the potential role of CHM in managing EC patients with malnutrition. CHM prescription analysis was performed to disclose the core CHMs and propose the involvement of pharmacological pathways. The results of this investigation may be helpful in facilitating the management strategy and feasibility of CHM among EC patients with malnutrition, and may offer guidance and direction to clinicians for the treatment of these patients in the future.

## 2 Materials and methods

### 2.1 Data source

This study utilized data from Chang Gung Research Database (CGRD), which archives comprehensive electronic medical records from Chang Gung Memorial Hospital (CGMH) in Taiwan. CGRD encompasses patient demographics, diagnostic details for outpatient/emergency visits and admissions, prescribed medications, comorbidities, procedural interventions, nursing care, national health insurance reimbursements, laboratory data, and cancer registry (Shao et al., 2019). Specifically, the cancer registry in CGRD includes exhaustive information on dates of diagnosis, cancer stages, tumor sizes, dates of treatment modalities, types and dates of recurrence, and mortality dates (Lee et al., 2021). CGMH, the largest private hospital network in Taiwan, consists of nine medical institutes, offering coverage for approximately 20% and 34% of oncological and outpatient needs in Taiwan, respectively. This extensive coverage renders CGRD an ideal database for clinical research (Tsai et al., 2017; Chen et al., 2022a; Chen et al., 2022b; Lee et al., 2022).

Additionally, CGRD contains detailed records of CHM usage among patients at CGMH. All CHMs are classified into single herbs (SH) and herbal formulas (HF). HF are composed of SH in fixed proportions, as documented in traditional Chinese medicine (TCM) classics, such as Jia-Wei-Xiao-Yao-San, where the effects are the combined outcome of the 10 SH it contains. An SH refers to a single herb listed in the TCM pharmacopeia, for example, *Hedyotis diffusa* Willd, and SH typically exhibit fewer and more specific effects compared to HFs. In clinical practice, TCM practitioners can freely add SH to HF within a single prescription to mitigate potential adverse effects or to enhance the therapeutic benefits of HF. All CHMs are manufactured according to strict practices, which include stringent regulations to prevent renal or liver toxicity and contamination from substances like pesticides or heavy metals. All information regarding CHM used in Taiwan were obtained from the website of the Ministry of Health and Welfare (https://service.mohw.gov.tw/DOCMAP/CusSite/TCMLQueryForm.aspx).

### 2.2 Study population and ethical considerations

**FIGURE 1:**
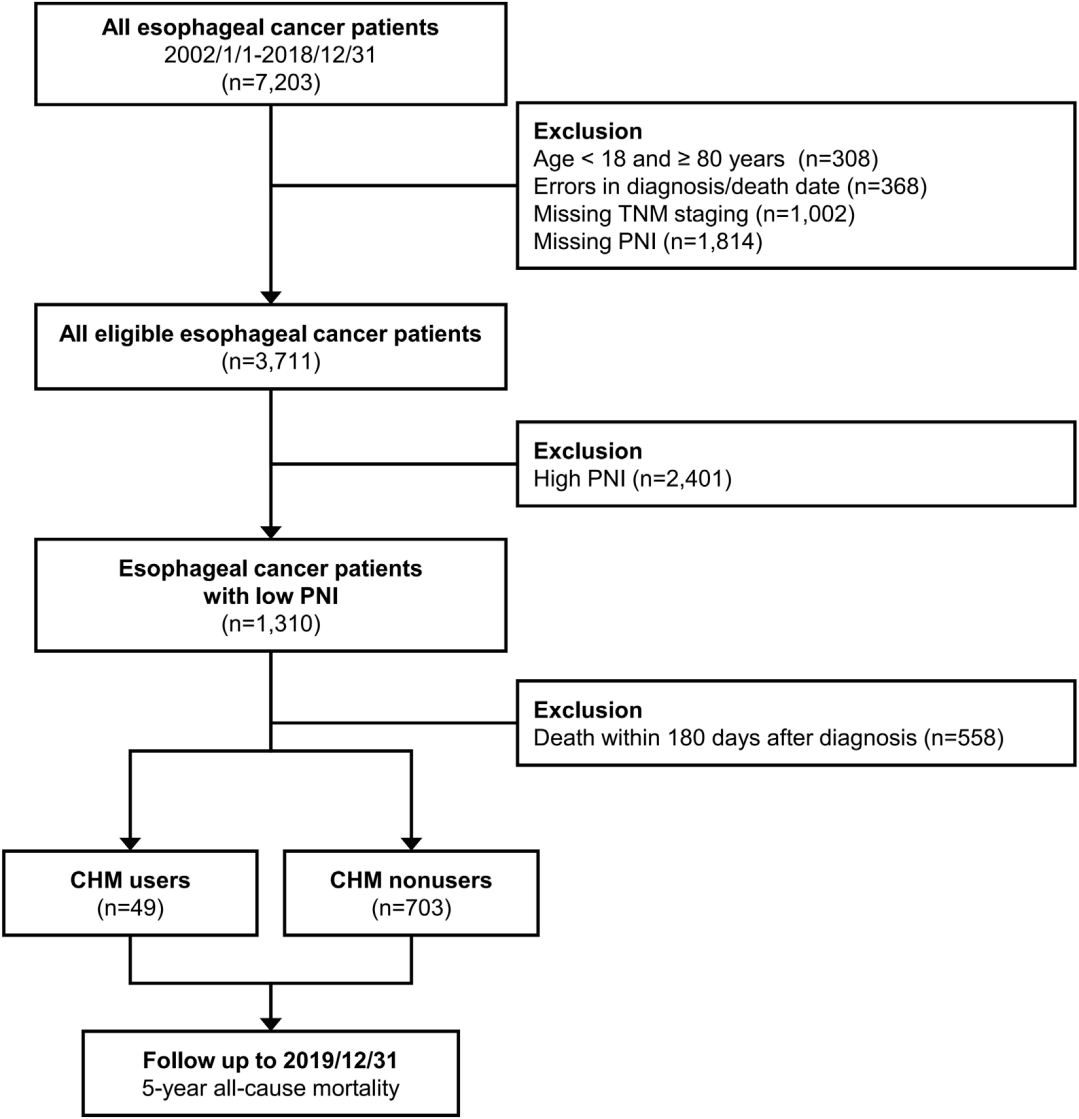
Flow diagram of this study. Abbreviations: CHM, Chinese herbal medicine; PNI, Prognostic Nutrition Index.

The International Classification of Diseases, 10th and Ninth Revision, Clinical Modification (ICD-9 and 10-CM) were used to determine the EC population. Patients diagnosed with EC (TNM staging, The American Joint Committee on Cancer versions 6 or 7; ICD-10-CM code: C15.0 -C16.2, ICD-9-CM code: 150) between January 2002 and December 2018 were included in the study. In contrast, patients aged <18 or ≥80 years, with errors in the diagnosis or death date, missing TNM staging records, missing PNI, or those who had died within 180 days from diagnosis were excluded. We first analyzed the OS among all patients with EC, and subsequently focused on those with PNI value < 45 (indicative of moderate to severe malnutrition). PNI is calculated based on the following equation (10 × serum albumin +0.005 × TLC). PNI <45 was also strongly correlated with diminished OS (Okadome et al., 2020). We classified the remaining eligible patients into two groups based on whether CHM was used after diagnosis. CHM users were defined as patients who received at least 1 treatment course with CHM during the study period. CHM nonusers were defined as those who did not receive treatment with CHM. The intention-to-treat design was applied to define CHM users and nonusers; therefore, it was not possible for patients to change group during the follow-up. The study design is shown in the flowchart (Figure 1). The Institutional Review Board of the Chang Gung Memorial Foundation in Taiwan approved the entire study protocol (approval number: 202300142B0C502). The provision of informed consent was deemed unnecessary due to the encryption of patient identifiers in the CGRD.

### 2.3 Outcome assessment and study covariates

All eligible patients were followed up until the occurrence of the primary endpoint, up to 5 years after diagnosis, or the end of 2019 (Figure 1). This study focused on the 5-year OS of EC patients with malnutrition, measured from the diagnosis date to death by any cause. Demographic and clinical covariates, including gender, age, body mass index (BMI), comorbidities, lifestyle factors, and pretreatment medications, were extracted from CGRD. EC-specific covariates, including cancer stage, tumor dimensions, and initial therapeutic approaches, were also collated. Biochemical profiles indicative of baseline physiological and nutritional status (e.g., serum albumin levels, hemoglobin levels, platelet-to-lymphocyte ratio, neutrophil-to-lymphocyte ratio, and PNI) were obtained (Yodying et al., 2016; Pirozzolo et al., 2019; Okadome et al., 2020). The most adverse laboratory findings within 1 year preceding the diagnosis of EC were recorded. Furthermore, the identification of comorbidities was based on at least two outpatient visits or a single inpatient admission for conditions including hypertension, type 2 diabetes mellitus, myocardial infarction, chronic obstructive pulmonary disease, cirrhosis, hepatitis B, hepatitis C, chronic kidney disease, and cerebrovascular diseases within 1 year prior to the diagnosis of EC. The Charlson Comorbidity Index was employed as a comprehensive measure of comorbidities and a predictive marker for OS (Kim et al., 2020; Kubo et al., 2021). Diagnosis codes utilized in this investigation are enumerated in Supplementary Material S1. In patients with EC, treatment modalities were diverse, including chemotherapy, radiotherapy, and surgical interventions for tumor resection (e.g., localized therapy and partial/total esophagectomy with or without gastrectomy). Baseline nutritional status was assessed using pretreatment serum albumin levels, hemoglobin levels, and BMI (Lim et al., 2017). The roles of platelet-to-lymphocyte ratio and neutrophil-to-lymphocyte ratio as systemic inflammation markers and prognostic indicators in EC were also investigated (Yodying et al., 2016).

### 2.4 Bias assessment

To mitigate confounding bias, we matched CHM users and nonusers through propensity score-based (PS-based) models as sensitivity tests. The real-time nature of CGRD, involving the real-time collection of data from daily clinical practices at CGMH, effectively eliminates recall bias. Furthermore, the immortal time bias may raise a concern about results estimation due to the lack of specific guidelines for initiating CHM treatment in EC patients. The various intervals between diagnosis and CHM treatments may be erroneously considered part of a treatment’s efficacy since EC patients may be classified as CHM nonusers due to their short life span and inability to receive CHM treatment. The immortal bias may lead to a potential influence on the interpretation of results. For this reason, we carefully delineated periods in our research. Consequently, we excluded from our analysis any patients who died within the first 180 days of follow-up. This exclusion criterion was established by calculating the median time from diagnosis to the commencement of CHM treatment. Integration of CGRD data with those of the national death registry database, supported by the National Health Informatics Project, facilitates accurate tracking of patient outcomes, thereby obviating registration and removing detection biases concerning mortality data.

### 2.5 Statistical analysis

This study involved an outcome evaluation and a Chinese herbal medicine network (CMN) analysis based on prescriptions of CHM for these patients. Baseline demographics were presented as means with standard deviation (SD) for continuous variables and frequencies with percentages for categorical variables. Differences between CHM users and nonusers were assessed using Student’s t-test and Chi-Square tests. Propensity score (PS) with overlap weighting, calibrated for age, gender, comorbidities, BMI, and initial treatments, were employed to balance the baseline status between the two cohorts and address imbalances in case numbers (Li et al., 2018). These covariates were used to generate the probability of using CHM as PS; PS and 1-PS were assigned to weight CHM nonusers and users, respectively (Thomas et al., 2020). OS estimates were derived using the Kaplan-Meier method, and hazard ratio (HR) for all-cause mortality was computed using the Cox proportional hazards regression model. Adjusted HR (aHR) considered all pertinent covariates other than those employed for PS generation. Multivariate Cox proportional hazards regression, stratified by demographic factors and sensitivity tests using diverse models, reinforced the association between CHM usage and OS. PS-based models for sensitivity tests and subgroup analyses included varying PS weights and matching methods, such as average treatment effect, average treatment effect on the treated patients, overlap weighting, and kernel matching (Li et al., 2018). Additional models, based on varying populations, included all patients without landmark analysis and 90-day landmark analysis.

Moreover, core CHMs and possible pharmacologic mechanisms were identified through network pharmacology analysis. Initially, CMN was constructed to visually articulate the treatment principles and identify the core CHMs for EC. The methodology for assembling the CMN has been comprehensively demonstrated in our previous research, and this approach has been commonly used to explore core proteins within complicated protein-protein networks (Jeong et al., 2001; Chen et al., 2015; Guo et al., 2021). Briefly, we used association rule mining to determine prevalent combinations of CHMs, and graphically rendered and scrutinized the CMN using social network analysis. The CHMs were clustered based on their interrelations. Core CHMs were characterized by their high frequency of use and extensive connections within the network, signifying their concurrent prescription with other CHMs. Furthermore, the specific targets of each core CHM were analyzed to evaluate the molecular pathways influenced by CHM in EC patients with malnutrition. This involved a Gene Ontology and pathway over-representation analysis utilizing data from the Reactome database (Wu et al., 2010; Fabregat et al., 2017; Fabregat et al., 2018). The underlying hypothesis for the over-representation analysis posits that if a molecular pathway is pertinent, the proteins associated with this pathway should be present at a higher frequency than what would be expected by random chance. To determine the statistical significance of each identified pathway, the false discovery rate was computed using the Benjamini–Hochberg method. Pathways with a false discovery rate ≤0.05 were considered statistically significant. This analysis was conducted separately across 6 distinct clusters of CHMs. Furthermore, the open-source platform KNIME was utilized for database management, along with an application programming interface. This ensured access to the latest information regarding the interplay between CHM compounds and their molecular targets, and facilitated the pathway enrichment analysis (Berthold et al., 2009; Chen et al., 2015; Wu et al., 2021; Chen et al., 2022b). Additionally, Stata Statistical Software (release 16; StataCorp LLC, College Station, TX, United States) and NodeXL were employed to conduct statistical analysis and the CMN analysis in this study. The analysis revealed the core CHMs amidst the intricate web of CMN. *p*-values < 0.05 were assumed to indicate statistically significant differences.

## 3 Results

### 3.1 Baseline demographic characteristics

From 1 January 2002 to 31 December 2018, 3,711 patients with EC were included in our investigation. In the terminal phase of our study, a focused analysis was conducted on 752 patients with EC exhibiting low PNI (<45), comprising 49 CHM users and 703 nonusers. Table 1 delineates the baseline demographic characteristics of EC patients with malnutrition. The comparative evaluation between the cohorts did not reveal statistically significant disparities in variables such as gender, age, BMI, comorbidities, lifestyle patterns, tumor dimensions, initial therapeutic interventions, and nutritional status (e.g., platelet-to-lymphocyte ratio and neutrophil-to-lymphocyte ratio). In terms of tumor stage, more than half of the patients in both groups were classified as advanced (TNM stage 3 and 4). Regarding the choice of treatment, about half of the patients in each group underwent radiotherapy and chemotherapy, with approximately 80% of them not undergoing tumor resection surgery. Most patients were male (95.9% and 94.3% of CHM users and nonusers, respectively). The mean age of patients was 54.7 years (SD: 10.0 years) and 56.3 years (SD: 10.3 years) for CHM users and nonusers, respectively. A majority of the patients in both groups were aged 41–60 years, accounting for 62.6% of the total eligible population. With respect to comorbid conditions, statistical analysis did not reveal significant differences in the prevalence of diabetes mellitus, hypertension, myocardial infarction, chronic obstructive pulmonary disease, cerebrovascular diseases, peripheral vascular disease, hepatitis B virus, hepatitis C virus, liver cirrhosis, and the Charlson Comorbidity Index between the two groups.

**TABLE 1:** Baseline characteristics of patients with EC at high risk of malnutrition.

	All patients (n = 752)	CHM nonusers (n = 703)	CHM users (n = 49)	*p*
**Demographics**				
**Gender**				0.64
Female	42 (5.6%)	40 (5.7%)	2 (4.1%)	
Male	710 (94.4%)	663 (94.3%)	47 (95.9%)	
**Age (years)**	56.2 (10.3)	56.3 (10.3)	54.7 (10.0)	0.29
**Age group**				0.67
≤40 years	39 (5.2%)	36 (5.1%)	3 (6.1%)	
41–60 years	471 (62.6%)	438 (62.3%)	33 (67.3%)	
>60 years	242 (32.2%)	229 (32.6%)	13 (26.5%)	
**BMI**	21.0 (4.0)	21.0 (3.9)	21.4 (4.9)	0.67
**Comorbidities**				
DM	61 (8.1%)	59 (8.4%)	2 (4.1%)	0.29
Hypertension	100 (13.3%)	93 (13.2%)	7 (14.3%)	0.83
MI	4 (0.5%)	4 (0.6%)	0 (0.0%)	0.60
COPD	55 (7.3%)	53 (7.5%)	2 (4.1%)	0.37
CVD	16 (2.1%)	14 (2.0%)	2 (4.1%)	0.33
PVD	3 (0.4%)	3 (0.4%)	0 (0.0%)	0.65
Hepatitis B virus	37 (4.9%)	33 (4.7%)	4 (8.2%)	0.28
Hepatitis C virus	27 (3.6%)	24 (3.4%)	3 (6.1%)	0.32
Liver cirrhosis	126 (16.8%)	120 (17.1%)	6 (12.2%)	0.38
CKD	20 (2.7%)	18 (2.6%)	2 (4.1%)	0.52
CCI	9.8 (2.1)	9.9 (2.1)	9.5 (2.4)	0.27
**Lifestyles**				
Cigarette smoking	293 (85.7%)	276 (85.4%)	17 (89.5%)	0.63
Alcohol consumption	294 (85.7%)	276 (85.4%)	18 (90.0%)	0.57
Betel nut chewing	206 (60.1%)	195 (60.4%)	11 (55.0%)	0.63
**Tumor size (mm)**	44.0 (37.2)	44.4 (37.4)	38.9 (36.0)	0.42
**TNM Stage**				0.51
**0**	14 (1.9%)	14 (2.0%)	0 (0.0%)	
I	80 (10.6%)	72 (10.2%)	8 (16.3%)	
II	107 (14.2%)	102 (14.5%)	5 (10.2%)	
III	335 (44.5%)	314 (44.7%)	21 (42.9%)	
IV	216 (28.7%)	201 (28.6%)	15 (30.6%)	
**Initial treatment**				
Surgery				0.30
No surgery for tumor resection	615 (81.8%)	576 (81.9%)	39 (79.6%)	
Local therapy	19 (2.5%)	18 (2.6%)	1 (2.0%)	
Partial esophagectomy	32 (4.3%)	32 (4.6%)	0 (0.0%)	
Total esophagectomy	2 (0.3%)	2 (0.3%)	0 (0.0%)	
Esophagectomy with laryngectomy/gastrectomy	78 (10.4%)	69 (9.8%)	9 (18.4%)	
Other surgery	6 (0.8%)	6 (0.9%)	0 (0.0%)	
Chemotherapy	410 (54.5%)	383 (54.5%)	27 (55.1%)	0.93
Radiotherapy	372 (49.5%)	345 (49.1%)	27 (55.1%)	0.41
**Biochemical profiles**				
Albumin (g/dL)	3.3 (0.5)	3.3 (0.5)	3.3 (0.5)	0.78
Hemoglobins (g/dL)	12.0 (2.2)	12.0 (2.1)	11.8 (2.4)	0.63
Lymphocyte (10^3^/µL)	1.3 (0.6)	1.3 (0.6)	1.3 (0.6)	0.68
ALT (U/L)	23.8 (24.0)	23.8 (24.6)	23.9 (14.1)	0.97
Total cholesterol (mg/dL)	158.8 (41.6)	157.5 (40.1)	173.2 (54.0)	0.071
PLR	237.7 (208.5)	236.3 (209.6)	256.9 (192.2)	0.51
NLR	6.2 (6.7)	6.2 (6.8)	6.2 (5.1)	0.98
PNI	39.4 (4.8)	39.4 (4.8)	39.1 (5.2)	0.60

Abbreviations: ALT, alanine aminotransferase; BMI, body mass index; CCI, charlson comorbidity index; CHM, chinese herbal medicine; CKD, chronic kidney disease; COPD, chronic obstructive pulmonary disease; CVD, cerebrovascular disease; DM, diabetes mellitus; EC, esophageal cancer; MI, myocardial infarction; NLR, neutrophil-to-lymphocyte ratio; PLR, platelet-lymphocyte ratio; PNI, prognostic nutrition index; PVD, peripheral vascular disease; TLC, total lymphocyte count.

Among patients with EC characterized by low PNI, a significant proportion was diagnosed with more advanced-stage disease (stage III: 44.5%, n = 335; stage IV: 28.7%, n = 216). The predominant initial therapeutic approaches consisted of chemotherapy and radiotherapy (54.5% and 49.5% of patients, respectively). Conversely, a mere 18.3% of patients in this cohort underwent surgical intervention as initial treatment. Statistical analysis did not reveal significant differences in the distribution of these treatment strategies between the two groups.

### 3.2 Use of CHM was associated with higher 5-year OS in EC patients with moderate to severe malnutrition

**FIGURE 2:**
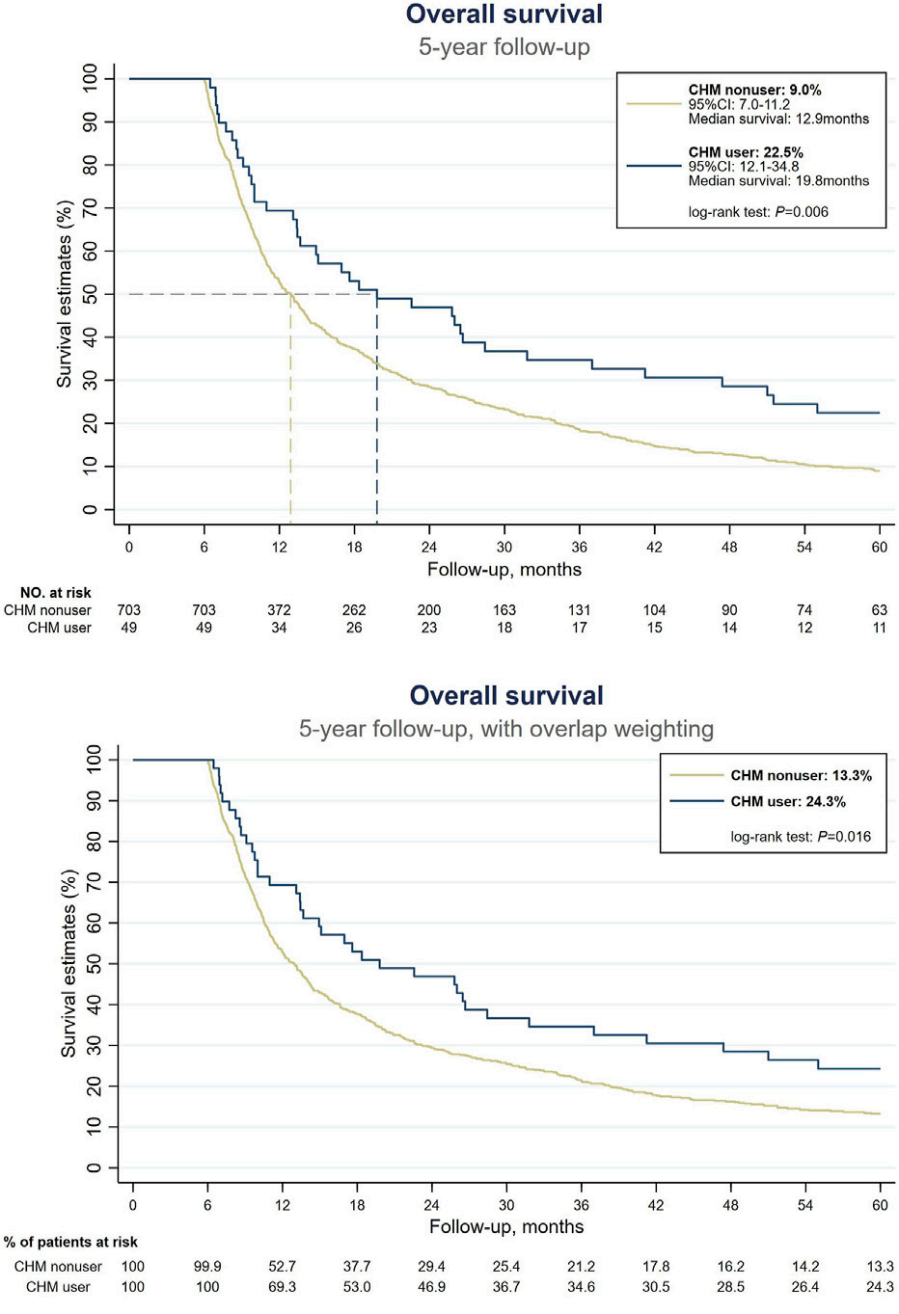
Survival analysis among EC patients with malnutrition. (Above) Without overlap weighting. (Below) With overlap weighting. Abbreviations: CHM, Chinese herbal medicine; EC, esophageal cancer.

Prior to conducting our survival analysis in EC patients with malnutrition, we examined the survival rates among all patients with EC. Our findings indicated that CHM users were associated with a higher probability of 5-year OS compared with CHM nonusers (Supplementary Material S2). In our primary focus group of EC patients with malnutrition, 678 of the 752 patients had expired at the end of the 5-year follow-up period. Among the survivors, 11 were CHM users and 63 were nonusers. Under the landmark design, CHM users were associated with a higher probability of 5-year OS than CHM nonusers (without overlap weighting: 22.5% vs. 9%; with overlap weighting: 24.3% vs. 13.3%, log-rank test: *p* = 0.006 and 0.016, respectively) (Figure 2). The median OS was observed to be 19.8 months, in stark contrast to the 12.9 months recorded in the nonuser group (Figure 2). The HR analysis indicates that CHM users exhibited a 31% decrement in the risk of all-cause mortality in comparison with nonusers (HR: 0.69, 95% confidence interval [CI]: 0.50–0.94, *p* = 0.019) (Table 2). An extensive examination incorporating all pertinent covariates revealed that CHM users experienced a 67% reduced risk in all-cause mortality compared with nonusers (aHR: 0.33, 95% CI: 0.14–0.76, *p* = 0.009). Additionally, a duration-dependent trend was evident in the CHM user group; longer duration of usage was correlated with progressively lower all-cause mortality risk (aHR: 0.44, 95% CI: 0.27–0.70, *p* = 0.001) (Table 2). Furthermore, Table 3 illustrates that the correlation between CHM utilization and reduced risk of all-cause mortality remained consistent across various models and sampled populations of EC patients with malnutrition, as evidenced in the sensitivity and subgroup analyses (*p* < 0.05). These diverse models consistently demonstrated that patients utilizing CHM exhibited significantly better survival outcomes compared with those who did not use CHM.

**TABLE 2:** Risk of all-cause mortality among malnourished EC patients with different durations of CHM use.

	HR (95% CI)	*p*	aHR (95% CI)	*p*
All CHM users (n = 49)	0.69 (0.50–0.94)	0.019	0.33 (0.14–0.76)	0.009
CHM nonusers (n = 703)	1 (reference)			
Use of CHM by duration				
≤21 days (n = 25)	0.99 (0.67–1.46)	0.961	1.08 (0.41–2.83)	0.875
>21 days (n = 24)	0.44 (0.27–0.70)	0.001	0.18 (0.07–0.51)	0.001

Abbreviations: CHM, chinese herbal medicine; CI, confidence interval; EC, esophageal cancer; HR, hazard ratio; aHR, adjusted hazard ratio.

**TABLE 3:** Sensitivity and subgroup analysis for the estimation of overall survival after CHM use.

	HR (95% CI)	*p*
Different PS models		
IPTW (n = 752)	0.69 (0.51–0.95)	0.025
ATT (n = 752)	0.67 (0.49–0.93)	0.016
Overlap weighting (n = 752)	0.68 (0.49–0.93)	0.016
1:5 PSM (n = 524)	0.70 (0.50–0.98)	0.035
Different populations		
All patients, without landmark design (n = 1,310)	0.54 (0.40–0.73)	0.000
Model with 90-day landmark analysis (n = 1,024)	0.60 (0.44–0.81)	0.001

Abbreviations: ATT, average treatment effect for the treated; CHM, chinese herbal medicine; CI, confidence interval; HR, hazard ratio; IPTW, inverse probability of treatment weighting; PSM, propensity score matching.

### 3.3 CMN for EC patients with low PNI

**FIGURE 3:**
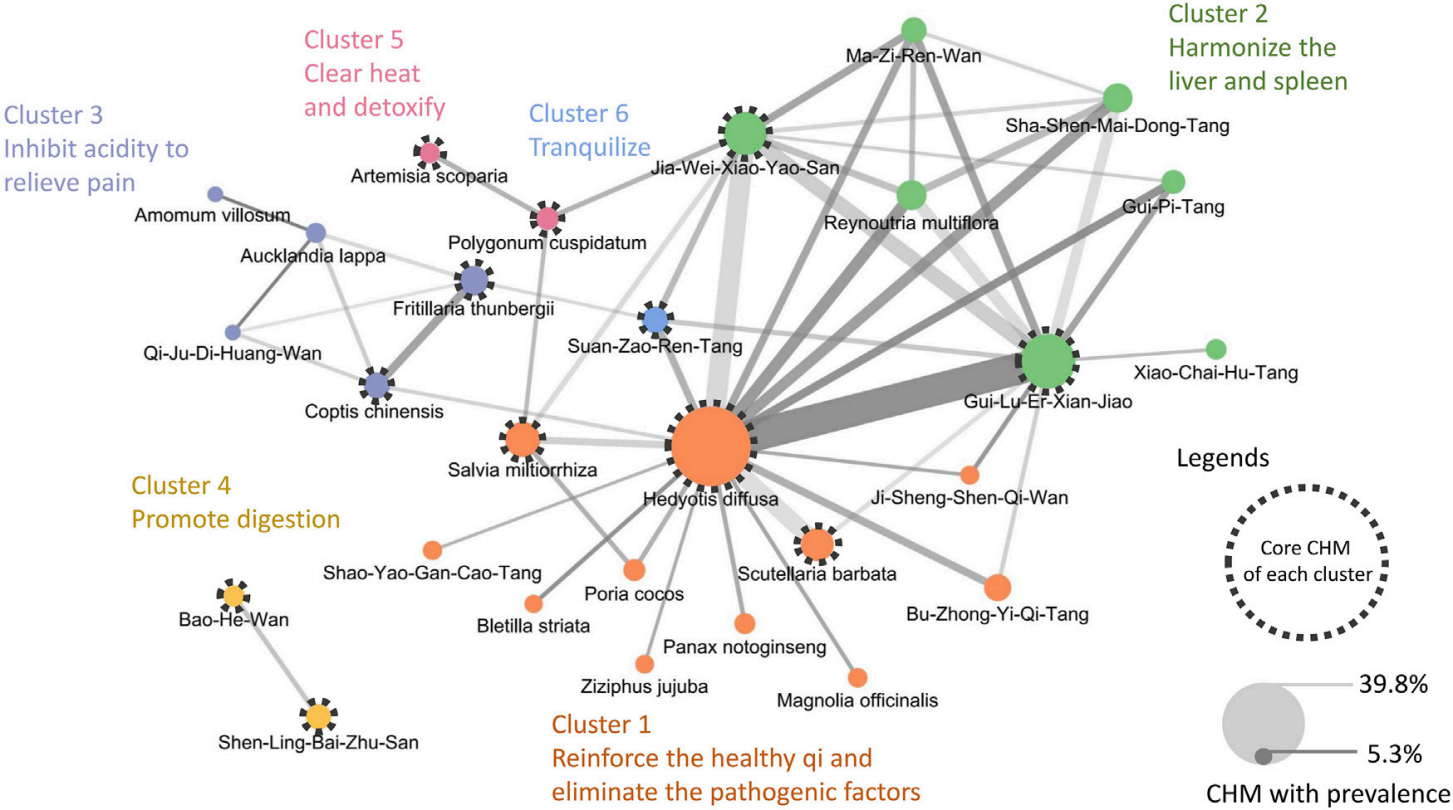
CMN of prescriptions for EC patients with malnutrition The width of connecting lines indicates the prevalence of each combination, and the color intensity indicates the confidence. A wider line denotes a higher prescription frequency of the CHM–CHM combination, while a darker line denotes a stronger relationship between connected CHMs. The size of a circle indicates the prevalence of each CHM, with larger circles denoting higher prevalence of CHMs. Abbreviations: CHM, Chinese herbal medicine; CMN, CHM network; EC, esophageal cancer.

This study revealed 665 prescriptions were made during the study period, involving 306 CHMs; the average number of CHMs used in each prescription was 6.9 (SD: 3.0). Table 4 lists the top 10 most commonly used CHMs. The most common CHM was *H. diffusa* Willd (39.8%), followed by Gui-Lu-Er-Xian-Jiao (25.9%), and Jia-Wei-Xiao-Yao-San (19.2%). The top 50 most common CHM–CHM combinations were used to construct the CMN (Supplementary Material S3), and social network analysis revealed the core CHMs (Figure 3). Table 5 shows the top 5 most commonly used CHM combinations. After clustering, the CHMs of each cluster are listed in Supplementary Material S4, and the composition of HF in the network is listed in Supplementary Material S5. Larger circles denote higher prevalence of CHMs in the CMN, wider connecting lines represent higher prescription frequency, and darker connection lines indicate stronger relationships between connected CHMs. The core CHMs among the 6 clusters could be identified based on their relatively high prevalence and more connections to other CHMs within clusters. By integrating CHM indications from CHM pharmacopeia into clustered CMN, we could determine the CHM features of each cluster. These included reinforcement of the healthy qi and elimination of pathogenic factors, harmonization of the liver and spleen, inhibition of acidity to relieve pain, promotion of digestion, clearance of heat and detoxification, and tranquilization (Figure 3).

**TABLE 4:** Top 10 CHMs prescribed for EC patients with malnutrition (prescriptions, n = 665).

CHM	Count	Prevalence (%)
*Hedyotis diffusa* Willd	265	39.8
Gui-Lu-Er-Xian-Jiao	172	25.9
Jia-Wei-Xiao-Yao-San	128	19.2
*Salvia miltiorrhiza* Bunge	98	14.7
*Scutellaria barbata* D. Don	90	13.5
*Reynoutria multiflora* (Thunb.) Moldenke	79	11.9
*Fritillaria thunbergii* Miq	78	11.7
Sha-Shen-Mai-Dong-Tang	77	11.6
Bu-Zhong-Yi-Qi-Tang	68	10.2
Suan-Zao-Ren-Tang	67	10.1

Abbreviations: CHM, chinese herbal medicine; EC, esophageal cancer.

**TABLE 5:** Top 5 CHM–CHM combinations for EC with malnutrition.

CHM A	CHM B	Prevalence (%)	Confidence	Lift
*Hedyotis diffusa* Willd	Gui-Lu-Er-Xian-Jiao	21.5	83.1	2.1
*Hedyotis diffusa* Willd	Jia-Wei-Xiao-Yao-San	14.6	75.8	1.9
*Hedyotis diffusa* Willd	*Scutellaria barbata D.* Don	12.6	93.3	2.3
Jia-Wei-Xiao-Yao-San	Gui-Lu-Er-Xian-Jiao	11.1	43	2.2
*Hedyotis diffusa* Willd	*Reynoutria multiflora (Thunb.)* Moldenke	9.0	75.9	1.9
Gui-Lu-Er-Xian-Jiao	*Reynoutria multiflora (Thunb.)* Moldenke	8.9	74.7	2.9

Prevalence indicates the percentage of each combination among all prescriptions (n = 665). Confidence and lift represent the strength of each combination, with higher values representing stronger connections between CHMs.

Abbreviations: CHM, chinese herbal medicine; EC, esophageal cancer.

### 3.4 Proposed pharmacologic pathways according to the core CHMs

**FIGURE 4:**
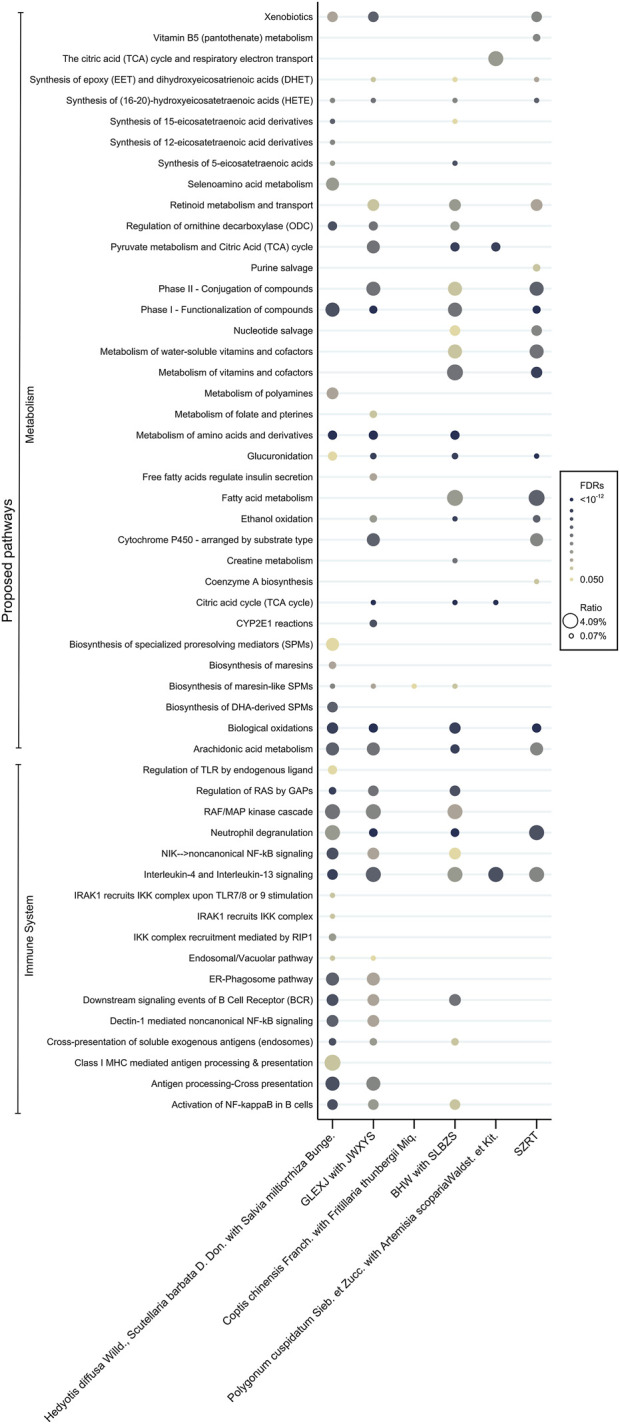
Proposed pharmacologic pathways according to the core CHMs. Abbreviations: CHM, Chinese herbal medicine.

The potential molecular pathways involved in the effects of the core CHMs within 6 clusters were postulated based on their associated binding proteins (refer to Figure 4 and Supplementary Material S6 for details). Our findings indicate a significant role of these CHMs in mechanisms related to the immune system and metabolic pathways, particularly in Clusters 1, 2, and 4. Cluster 1 (reinforcement of healthy qi and elimination of pathogenic factors) and Cluster 2 (harmonization of the liver and spleen) exhibited the most substantial involvement in immune regulation, featuring 17 and 12 pathways, respectively. Cluster 4 (promotion of digestion) showed the greatest involvement in metabolic regulation, with a total of 21 pathways. In contrast, Cluster 3 (inhibition of acidity to relieve pain) and Cluster 5 (clearance of heat and detoxification) demonstrated a relatively lower association with immune and metabolic processes.

## 4 Discussion

The present study evaluated the impact of CHMs on the survival of EC patients with moderate to severe malnutrition. Advanced tumors and poor nutritional status could potentially lead to variations in the prognosis; however, there were no statistical differences between CHM users group and nonusers group, indicating that the impact can be considered negligible. CHM users showed higher 5-year OS rates (22.5% without overlap weighting, 24.3% with overlap weighting) compared with nonusers (9% and 13.3%, respectively). CHM users had a significantly longer median OS than nonusers (19.8 vs. 12.9 months, respectively). HR analysis indicated a 31% risk reduction in all-cause mortality for CHM users compared with nonusers. In addition, aHR analysis revealed a 67% reduction in all-cause mortality risk among CHM users.

This is the first study of the long-term effects of CHMs on the survival of EC patients with malnutrition. Previous research has suggested that CHM has the potential to enhance the quality of life for patients with EC undergoing radiotherapy or chemotherapy, as well as ameliorate certain adverse events associated with such treatments (Yang et al., 2012; Yeh et al., 2014; Chen et al., 2016; Hsu et al., 2016). However, existing CHM studies have seldom focused on the malnourished population among patients with EC. The physical condition of these patients frequently results in an inability to complete the requisite oncological therapies, consequently leading to particularly poor prognosis (Li et al., 2020; Okadome et al., 2020; Qiu et al., 2020; Jiang et al., 2021). The nutritional status of a patient has a more significant impact in certain tumor types, such as EC and other gastrointestinal cancers, as well as head and neck malignancies. It reflects the complex interplay between tumor burden, inflammatory states, reduced caloric intake, and malabsorption (Suzuki et al., 2013; Bossi et al., 2021; Nishikawa et al., 2021). This paper serves as a critical augmentation to the existing body of literature, addressing this deficiency in research. The findings suggest that prolonged utilization of CHM improves OS for EC patients with malnutrition. Therefore, CHM could be considered as an adjunct therapy for the entire cohort of patients with EC, especially those suffering from malnutrition.

The Gastroenterological Society of Taiwan’s Consensus Statement on Nutrition Therapy in patients with EC recommends that preoperative nutritional support (e.g., tube feeding) for at least 7–10 days should be considered for patients at high risk of malnutrition (Chen et al., 2018). Further studies have demonstrated that nutritional interventions can significantly improve OS (Cox et al., 2016). Therefore, nutritional intervention is also mentioned in official treatment recommendations (Chen et al., 2018). Despite these advancements, the prognosis for EC remains poor. Considering that CHM can enhance nutritional absorption through different mechanisms mentioned in this article, future inclusion of CHM in current clinical treatment protocols, in conjunction with nutritional therapy, could result in further improvements.

To understand the mode through which CHM ameliorates the OS of EC patients with malnutrition, we analyzed relevant prescriptions dispensed to patients. *Hedyotis diffusa* Willd. emerged as the predominant CHM, prescribed in approximately 40% of cases. This prevalence is consistent with our previous findings in patients with stage IV EC (Chen et al., 2022b), underscoring the integral role of *H. diffusa* Willd. in the therapeutic regimen for EC. Nevertheless, a notable difference was observed in the second and third most prevalent prescriptions, namely, Gui-Lu-Er-Xian-Jiao and Jia-Wei-Xiao-Yao-San, respectively. Such variation exemplifies the quintessence of the “identification/syndrome differentiation and treatment” approach of TCM, a hallmark of the diagnostic and therapeutic philosophy of TCM that advocates “different treatments for the same disease” based on individualized patient assessments.

Due to the complexity of disease manifestations and the distinctive therapeutic characteristics of CHM, the prescriptions for EC patients with malnutrition appear to be diverse. This diversity emphasizes the importance of network pharmacology analysis in CHM. Through an examination of the interconnections among CHM, we identified 6 distinct clusters of CHMs and summarized their characteristics according to the pharmacological effects. Among these pharmacological effects, several key benefits include reinforcement of the healthy qi (also known as immune enhancement), fortification of the spleen (also known as anti-cancer cachexia), invigoration of the stomach (also termed digestive function promotion) and elimination of pathogenic factors (also known as anti-tumor activity). Previous research has indicated that cancer-associated malnutrition is commonly linked to the two physiological mechanisms of immune dysfunction and digestive metabolic dysregulation (Suzuki et al., 2013; Nishikawa et al., 2021). In cancers pertaining to the swallowing function, such as EC, an additional element concerning digestive tract compression and obstruction emerges. These 3 aspects precisely align with the therapeutic approach of CHM when treating such patients. This may explain the observed enhancement in OS for EC patients with malnutrition.

Immune function may play a pivotal role in nutrient absorption. Prior research has established a link between cachexia and the aberrations in inflammatory and immune responses induced by tumors. Several cytokines, such as TNF-α, IL-1, IL-6, and IFN-γ, have been implicated in the pathogenesis of cancer-associated cachexia, particularly in the mediation of muscle proteolysis (Suzuki et al., 2013; Baracos et al., 2018; Nishikawa et al., 2021). Our study elucidates that CHMs prescribed for EC with malnutrition exert their influence within the proposed immunological pathway, particularly those in Cluster “reinforcement of healthy qi and elimination of pathogenic factors” and Cluster “harmonization of the liver and spleen”. Bu-Zhong-Yi-Qi-Tang (Cluster “reinforcement of healthy qi and elimination of pathogenic factors”) induces divergent modulations in T lymphocyte functionality, concomitantly diminishing the levels of IL-6; this evidence highlights its immunoregulatory mechanisms (Mori et al., 1999; Liu et al., 2019). *Panax ginseng* C.A. Meyer (PG), contained in Gui-Lu-Er-Xian-Jiao, has been noted for its capacity to regulate tumor-associated immune responses (Wang et al., 2020; Zhao et al., 2022) and has also been proved to mitigate the symptoms of cancer cachexia by reducing the levels of pro-inflammatory cytokines TNF-α and IL-6 (Lu et al., 2020).

Furthermore, cancer cachexia is intricately linked to digestive and metabolic dysregulation, characterized by diminished food intake and metabolic changes (Baracos et al., 2018). Our investigation revealed that CHMs for EC patients with malnutrition play a critical role in metabolism-related pathways, particularly those in Cluster “harmonization of the liver and spleen” and Cluster “promotion of digestion”. For example, the third-ranked Jia-Wei-Xiao-Yao-San (Cluster “harmonization of the liver and spleen”) ameliorates symptoms in patients with functional dyspepsia and adjusts abnormal gastrointestinal functions (Qu et al., 2010; Chen et al., 2020). Studies on Shen-Ling-Bai-Zhu-San (Cluster “promotion of digestion”) revealed efficacy in mitigating histological damage to the colon and reversing pathological alterations in tight junctions and microvilli within the intestinal tract, thereby enhancing digestive metabolism (Ji et al., 2019; Qu et al., 2023).

Finally, CHM exhibits direct anti-cancer properties. It has the potential to delay the progression of tumor-induced compression, consequently postponing the impact on swallowing function. *Hedyotis diffusa* Willd. is renowned for its anti-cancer effects (Chen et al., 2008; Ye and Huang, 2012; Tao and Balunas, 2016), the extract of *Scutellaria barbata* D. Don induces apoptosis and inhibits autophagic processes within malignant cellular lines (Zhang et al., 2017; Liu et al., 2022), and PG can exerts anti-tumor effects on gastrointestinal tract tumors through multiple pathways (Ni et al., 2022).

However, this study has several limitations. Firstly, CGRD only encompasses CHM prescriptions issued by CGMH. Consequently, patients with EC receiving CHM or alternative therapies at local clinics or other medical institutions are not definitively accounted for, potentially leading to an underestimation of CHM usage. Secondly, in patients with advanced-stage EC, severe dysphagia may adversely impact the nutritional status and prognosis, introducing a potential selection bias and possibly inflating the effect size observed among CHM users. It is noteworthy that enteral feeding, rather than the parenteral route, is commonly preferred for nutritional support in patients with EC. Treatments with CHMs are compatible with various forms of enteral feeding (Bozzetti, 2010). Given that most patients with EC have an established enteral feeding pathway, and there are negligible differences in baseline serum albumin levels, hemoglobin levels, and PNI between CHM users and nonusers. Thirdly, since CHM is not a conventional treatment modality for EC, fewer people opt for CHM treatment, resulting in a lower number of CHM users in this study (49 users vs. 703 nonusers). To address this, we employed PS with an overlap weighting technique to balance the potential differences in demographic features of CHM users and nonusers without further losing eligible cases. Additionally, the absence of standardized guidelines for the initiation of CHM treatment could lead to immortal time bias. To counter this, the use of landmark analysis in this study further reduces the risk. Lastly, it must be emphasized that our study was retrospective and observational in nature, constrained by the limitations inherent to the database used. Consequently, further extensive randomized controlled trials focusing on specific SH or HF components are imperative to establish the concrete causal relationships between various CHM prescriptions and clinical outcomes.

## 5 Conclusion

The results of our study suggest a correlation between the utilization of CHM and enhanced clinical outcomes in patients suffering from EC with malnutrition. Additionally, our analysis through CMN identified core CHMs and combinations of formulations. These formulations play a pivotal role in immunomodulation and metabolic regulation. These findings may provide a foundation for more extensive clinical and fundamental research focused on the application of CHM in the management of malnutrition in patients with EC.

## Data Availability

The original contributions presented in the study are included in the article/Supplementary Material, further inquiries can be directed to the corresponding author.

## Supplementary material

SUPPLEMENTARY MATERIAL S1 Diagnosis codes used in this study.

SUPPLEMENTARY MATERIAL S2 Survival analysis among patients with esophageal cancer (EC).

SUPPLEMENTARY MATERIAL S3 Chinese herbal medicine (CHM) combinations used to construct the CHM network (CMN).

SUPPLEMENTARY MATERIAL S4 List of Chinese herbal medicines (CHMs) of each cluster.

SUPPLEMENTARY MATERIAL S5 Composition of herbal formula (HF) in the Chinese herbal medicine network (CMN).

SUPPLEMENTARY MATERIAL S6 Binding proteins of the core herb of the 6 clusters of Chinese herbal medicines (CHMs).
