# Supplementary material for: Can Chinese herbal medicine offer feasible solutions for newly diagnosed esophageal cancer patients with malnutrition? a multi-institutional real-world study
Source: Front Pharmacol. 2024 May 24;15:1364318. doi: 10.3389/fphar.2024.1364318 (PMC11157104; doi:10.3389/fphar.2024.1364318)
Supplement: Supplementary file 1 [file DataSheet1.zip › Supplementary Materials/Supplementary Material S5.DOCX]

Supplementary material S5. Composition of herbal formula (HF) in the Chinese herbal medicine network (CMN).

| Herbal formula (HF) | Composition |
| --- | --- |
| Gui-Lu-Er-Xian-Jiao | Lycium barbarum L. or Lycium chinense Mill., Chinemys reevesii (Gray), Panax ginseng C.A.Mey., Cervus elaphus Linnaeus or Cervus nippon Temminck |
| Jia-Wei-Xiao-Yao-San | Paeonia × suffruticosa Andrews, Gardenia jasminoides Ellis., Mentha canadensis L., Angelica sinensis (Oliv.) Diels, Bupleurum chinense DC., Glycyrrhiza uralensis Fisch. ex DC., Paeonia lactiflora Pall., Atractylodes macrocephala Koidz., Zingiber officinale Roscoe, Poria cocos (Schw.) Wolf |
| Sha-Shen-Mai-Dong-Tang | Morus alba L., Polygonatum odoratum (Mill.) Druce var. pluriflorum (Miq.) Ohwi, Adenophora tetraphylla (Thunb.) Fisch or Adenophora axilliflora Borb. or Glehnia littoralis F. Schmidt ex Miq., Glycyrrhiza uralensis Fisch. ex DC., Ophiopogon japonicus (Thunb.) Ker Gawl., Trichosanthes kirilowii Maxim. or Trichosanthes japonica Regel, Dolichos lablab L. |
| Bu-Zhong-Yi-Qi-Tang | Zizyphus jujuba Mill., Citrus reticulata Blanco, Angelica sinensis (Oliv.) Diels, Astragalus mongholicus Bunge or Astragalus membranaceus (Fisch.) Bge., Bupleurum chinense DC., Panax ginseng C.A.Mey., Glycyrrhiza uralensis Fisch. ex DC., Atractylodes macrocephala Koidz., Cimicifuga heracleifolia Kom. or Cimicifuga dahurica (Turcz.) Maxim. or Cimicifuga foetida L., Zingiber officinale Roscoe |
| Suan-Zao-Ren-Tang | Pulvis Talci, Glycyrrhiza uralensis Fisch. ex DC., Anemarrhena asphodeloides Bunge, Conioselinum anthriscoides 'Chuanxiong', Poria cocos (Schw.) Wolf, Ziziphus jujuba Mill. |
| Ma-Zi-Ren-Wan | Magnolia officinalis Rehder & E.H.Wilson or Magnolia officinalis var. biloba Rehder & E.H.Wilson., Citrus × aurantium L., Rheum palmatum L. or Rheum tanguticum (Maxim. ex Regel) Balf. or Rheum officinale Baill., Paeonia lactiflora Pall., Cannabis sativa L., Prunus armeniaca L. |
| Shen-Ling-Bai-Zhu-San | Amomum villosum Lour. or Amomum villosum var. xanthioides (Wall. ex Baker) T.L.Wu & S.J.Chen or Amomum longiligulare T.L. Wu, Zizyphus jujuba Mill., Panax ginseng C.A.Mey., Glycyrrhiza uralensis Fisch. ex DC., Platycodon grandiflorus (Jacq.) A.DC., Atractylodes macrocephala Koidz., Dioscorea oppositifolia L., Poria cocos (Schw.) Wolf, Coix lacryma-jobi L., Dolichos lablab L., Nelumbo nucifera Gaertn |
| Gui-Pi-Tang | Euphoria longan (Lour.) Steud., Zizyphus jujuba Mill., Angelica sinensis (Oliv.) Diels, Astragalus mongholicus Bunge or Astragalus membranaceus (Fisch.) Bge., Aucklandia lappa Decne. or Saussurea lappa Clarke, Panax ginseng C.A.Mey., Glycyrrhiza uralensis Fisch. ex DC., Polygala tenuifolia Willd. or Polygala sibirica L., Atractylodes macrocephala Koidz., Zingiber officinale Roscoe, Poria cocos (Schw.) Wolf, Ziziphus jujuba Mill. |
| Bao-He-Wan | Crataegus pinnatifida Bge. var. major N. E. Br. or Crataegus pinnatifida Bge., Forsythia suspensa (Thunb.) Vahl, Massa Fermentata Medicinalis, Citrus reticulata Blanco, Pinellia ternata (Thunb.) Makino, Poria cocos (Schw.) Wolf, Raphanus sativus L. |
| Ji-Sheng-Shen-Qi-Wan | Cinnamomum cassia (L.) J.Presl, Paeonia × suffruticosa Andrews, Cornus officinalis Siebold & Zucc., Achyranthes bidentata Blume, Aconitum carmichaeli Debeaux, Rehmannia glutinosa (Gaertn.) DC., Alisma plantago-aquatica L. or Alisma plantago-aquatica subsp. orientale (Sam.) Sam., Dioscorea oppositifolia L., Poria cocos (Schw.) Wolf, Plantago asiatica L. or Plantago depressa Willd. |
| Shao-Yao-Gan-Cao-Tang | Paeonia lactiflora Pall., Glycyrrhiza uralensis Fisch. |
| Xiao-Chai-Hu-Tang | Bupleurum chinense DC., Scutellaria baicalensis Georgi, Panax ginseng C.A.Mey., Glycyrrhiza uralensis Fisch., Zingiber officinale Roscoe, Pinellia ternata (Thunb.) Makino, Ziziphus jujuba Mill. |
| Qi-Ju-Di-Huang-Wan | Rehmannia glutinosa Libosch., Cornus officinalis Siebold & Zucc., Dioscorea polystachya Turcz., Alisma plantago-aquatica L. subsp. orientale (Sam.) Sam., Paeonia suffruticosa Andrews, Wolfiporia extensa (Peck) Ginns (Poria cocos (Schwein.) F.A.Wolf), Lycium chinense Mill., Chrysanthemum morifolium Ramat. |
