# Supplementary figures and images for: Can Chinese herbal medicine offer feasible solutions for newly diagnosed esophageal cancer patients with malnutrition? a multi-institutional real-world study
Source: Front Pharmacol. 2024 May 24;15:1364318. doi: 10.3389/fphar.2024.1364318 (PMC11157104; doi:10.3389/fphar.2024.1364318)

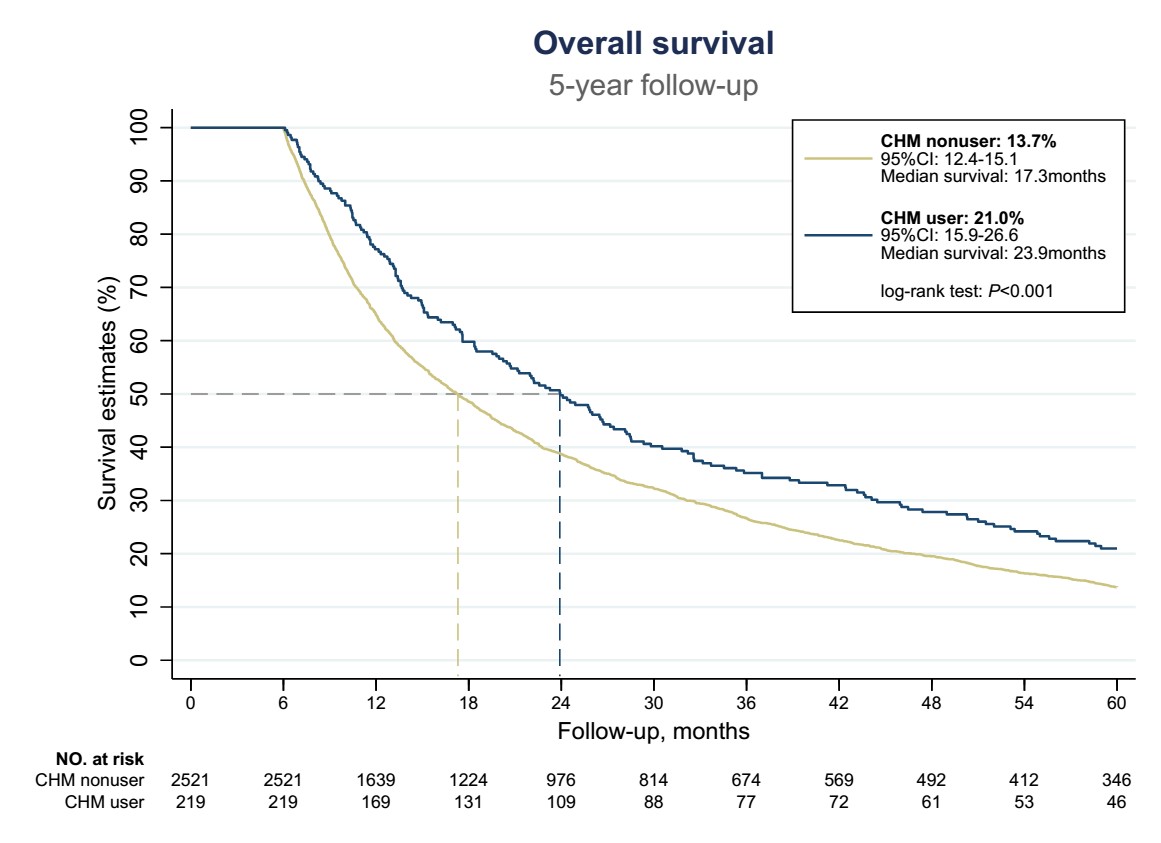

Supplement: Supplementary file 1 [file DataSheet1.zip › Supplementary Materials/Supplementary Material S2.JPEG]
